# AAV-compatible optogenetic tools for activating endogenous calcium channels in vivo

**DOI:** 10.1186/s13041-023-01061-7

**Published:** 2023-10-17

**Authors:** Yeon Hee Kook, Hyoin Lee, Jinsu Lee, Yeonji Jeong, Jaerang Rho, Won Do Heo, Sangkyu Lee

**Affiliations:** 1 Center for Cognition and Sociality, Institute for Basic Science (IBS), Daejeon, 34126 Republic of Korea; 2 Department of Bioscience and Biotechnology, Graduate School, Chungnam National University, Daejeon, 34134 Korea; 3 Department of Biological Sciences, Korea Advanced Institute of Science and Technology (KAIST), Daejeon, 34141 Republic of Korea; 4 KAIST Institute for the BioCentury, Korea Advanced Institute of Science and Technology (KAIST), Daejeon, 34141 Republic of Korea

**Keywords:** Calcium ion, Optogenetics, Adeno-associated virus, Neurons, Glial cells

## Abstract

Calcium ions (Ca^2+^) play pivotal roles in regulating diverse brain functions, including cognition, emotion, locomotion, and learning and memory. These functions are intricately regulated by a variety of Ca^2+^-dependent cellular processes, encompassing synaptic plasticity, neuro/gliotransmitter release, and gene expression. In our previous work, we developed ‘monster OptoSTIM1’ (monSTIM1), an improved OptoSTIM1 that selectively activates Ca^2+^-release–activated Ca^2+^ (CRAC) channels in the plasma membrane through blue light, allowing precise control over intracellular Ca^2+^ signaling and specific brain functions. However, the large size of the coding sequence of monSTIM1 poses a limitation for its widespread use, as it exceeds the packaging capacity of adeno-associated virus (AAV). To address this constraint, we have introduced monSTIM1 variants with reduced coding sequence sizes and established AAV-based systems for expressing them in neurons and glial cells in the mouse brain. Upon expression by AAVs, these monSTIM1 variants significantly increased the expression levels of cFos in neurons and astrocytes in the hippocampal CA1 region following non-invasive light illumination. The use of monSTIM1 variants offers a promising avenue for investigating the spatiotemporal roles of Ca^2+^-mediated cellular activities in various brain functions. Furthermore, this toolkit holds potential as a therapeutic strategy for addressing brain disorders associated with aberrant Ca^2+^ signaling.

**Supplementary Information:**

The online version contains supplementary material available at 10.1186/s13041-023-01061-7.

## Introduction

Calcium ions (Ca^2+^) serve as ubiquitous second messengers, playing critical roles in regulating a wide array of brain functions, including cognition, emotion, locomotion, and learning and memory [1–4]. Intracellular Ca^2+^ signaling is tightly regulated in space and time, and its aberrant regulation in the central nervous system has been associated with various neurological disorders, such as epilepsy, chronic pain, psychiatric diseases and neurodegeneration [5–9]. Brain cells possess a diverse molecular machinery, including channels and pumps localized within the plasma membrane and membranes of various subcellular organelles, that encode specific Ca^2+^ signals [9, 10]. The functional outcomes of Ca^2+^ signaling are highly dependent on intracellular contexts and signal parameters (e.g., amplitude, duration, and location) [11], with distinct input signals regulating specific neuronal functions, including synaptic plasticity, neurotransmitter release, and gene expression [7, 12]. In addition to its roles in neurons, Ca^2+^-mediated signaling is also critically involved in the regulation of various processes in glial cells, including gliotransmitter release, homeostasis, and immune responses [13–15].

To directly examine causal relationships between Ca^2+^ signals and cellular functions in space and time, we and others have previously developed a series of molecular optogenetic technologies designed to control intracellular Ca^2+^ levels through light stimulation [16]. These technologies primarily target CRAC (Ca^2+^-release–activated Ca^2+^) channels, which are widely expressed and highly selective for Ca^2+^ [10]. STIM1 (Stromal interaction molecule 1), a key regulator in the process of store-operated Ca^2+^ entry (SOCE), plays a key role in these technologies. Under natural conditions, STIM1, which senses Ca^2+^ levels in the ER lumen through domains in its N-terminus, resides in the endoplasmic reticulum (ER) membrane [10]. Upon depletion of ER Ca^2+^, STIM1 oligomerizes and undergoes conformational changes in its C-terminal domains. It subsequently translocates to the plasma membrane, where it binds to and activates CRAC channels, thereby leading to an increase in intracellular Ca^2+^ levels.

**Fig. 1:**
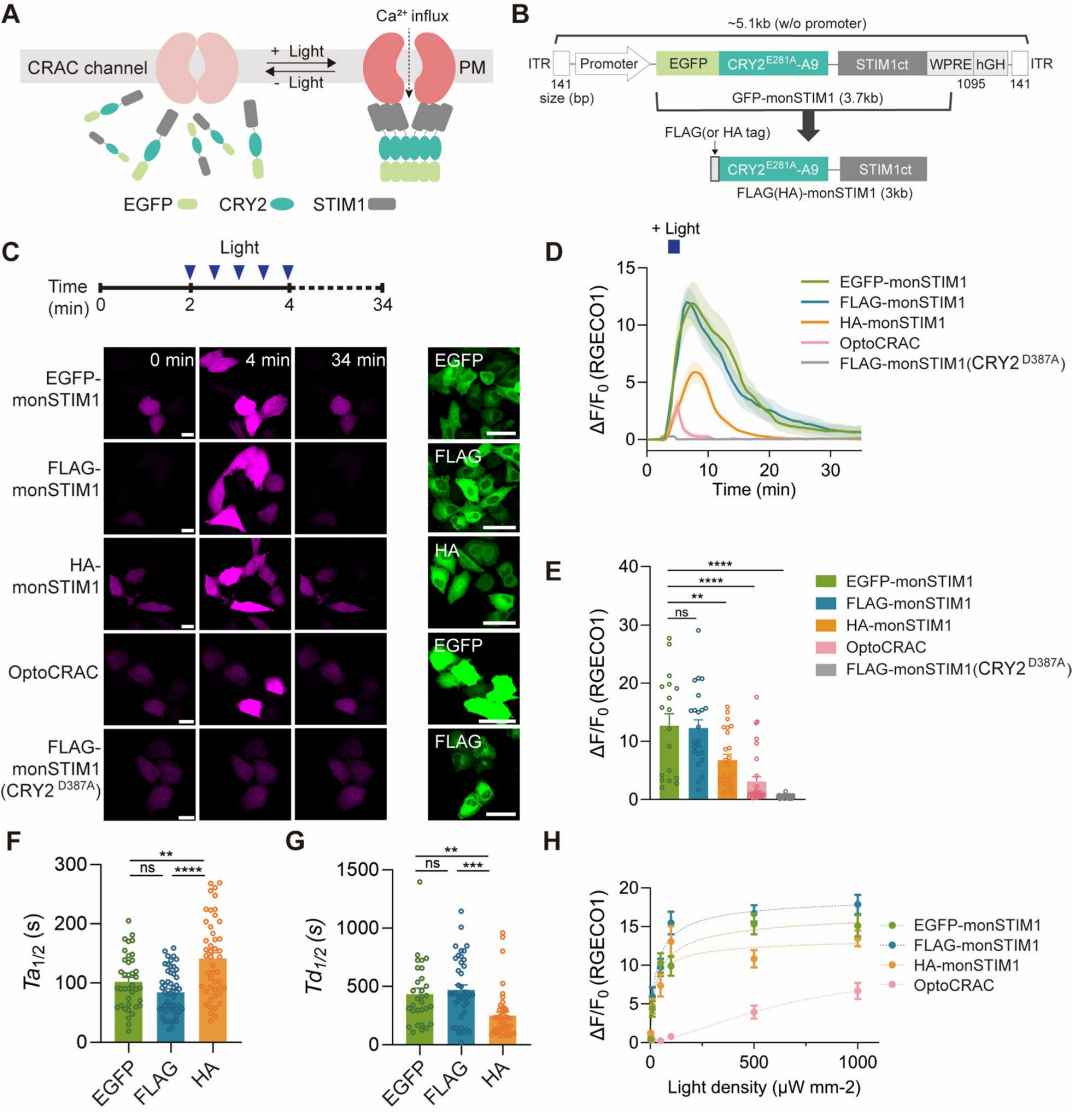
Reducing the size of monSTIM1 by replacing GFP with a small tag. **A** Schematic of the working mechanism of GFP-monSTIM1. **B** Sizes of coding sequences of monSTIM1 and components of the AAV cassette. **C** Fluorescence images of cells co-expressing RGECO1 and monSTIM1 variants or OptoCRAC. Blue light was delivered for 2 min at 30-s intervals, and changes in intracellular Ca^2+^ levels were monitored by imaging RGECO1 (left, magenta images). Expression of monSTIM1 variants and OptoCRAC (right, green images). **D** Summary data showing changes in the normalized intensity of RGECO1 over time upon transient activation of monSTIM1 variants and OptoCRAC. **E** Summary data showing maximum fold changes (ΔF/F_0_) in RGECO1 intensity for monSTIM1 variants and OptoCRAC upon blue light stimulation (EGFP-monSTIM1: n = 18; FLAG-monSTIM1: n = 23; HA-monSTIM1: n = 24; OptoCRAC: n = 31; FLAG-monSTIM1(CRY2^D387A^): n = 38 cells). **F**, **G** Quantification of activation **F** and deactivation **G** kinetics of monSTIM1 variants (EGFP-monSTIM1: n = 30–38; FLAG-monSTIM1: n = 42–50; HA-monSTIM1: n = 45–47 cells). **H** Summary data showing the light-sensitivity of each optogenetic module. Data are presented as means ± SEM (*p < 0.05, **p < 0.01, ***p < 0.001, ****p < 0.0001; Student two-tailed *t* test); ns, not significant (*p* > 0.05)

Two types of optogenetic actuators based on different photoreceptors have been developed for activation of CRAC channels [17]. The first type employs the LOV2 (light-oxygen-voltage-sensing 2) domain of a phototropin derived from *Avena Sativa*. Conjugating the LOV2 domain to the active STIM1 fragment introduces steric hindrance, silencing STIM1 activity in the absence of light [18–20]. Blue light stimulation induces conformational changes within the LOV2 domain that lead to its dissociation from the C-terminal Jα helix. This dissociation relieves STIM1 from inhibition, enabling the activation of CRAC channels and increasing intracellular Ca^2+^ levels. The second type of optogenetic actuator, termed OptoSTIM1 [21], utilizes cryptochrome 2 (CRY2) derived from *Arabidopsis thaliana*. Leveraging the light-mediated homo-interaction property of CRY2, the cytosolic fragment of STIM1, when conjugated with CRY2, undergoes homo-oligomerization upon light stimulation. Homo-oligomerized STIM1, in turn, translocates to the plasma membrane, where it activates CRAC channels, mimicking the natural action mechanism of STIM1 (Fig. 1A). OptoSTIM1 has proven effective in activating Ca^2+^ influx in various cell types and been shown to successfully control targeted brain functions [21]. Although LOV2-based tools possess certain advantages over OptoSTIM1, such as faster kinetics for (de)activation and smaller size, a comparative analysis revealed that the fold-change in Ca^2+^ increase achieved by OptoSTIM1 is superior to that of LOV2-based methods [16].

As part of our continuing efforts to engineer CRY2, we recently found that a CRY2(E281A) mutant significantly reduces the basal activity of OptoSTIM1 in the dark while simultaneously enhancing its sensitivity to blue light [22]. This previous study also revealed that the combination of CRY2(E281A) with C-terminal conjugation of 9 amino acids (A9) remarkably augments the efficiency of light-dependent oligomerization of CRY2. Building upon these discoveries, we have developed a second-generation version of OptoSTIM1, termed monster OptoSTIM1 (monSTIM1), that exhibits approximately a 55-fold higher sensitivity to blue light compared to the original version. Importantly, this advancement enables activation of Ca^2+^ signaling in the mouse brain in vivo through non-invasive illumination, eliminating the need for optic fibers.

However, combining the size of the coding sequence of monSTIM1 with AAV cassette components, including ITRs (inverted terminal repeats), WPRE (woodchuck hepatitis virus post-transcriptional regulatory element) and hGH (human growth hormone) polyadenylation signal results in a total sequence size of ~ 5.1 kb without a promoter, exceeding the packaging capacity (~ 5.0 kb) of adeno-associated viruses (AAVs) (Fig. 1B) [23]. Because of these size constraints, we utilized lentivirus in our previous studies to express optogenetic Ca^2+^ modulators in the mouse brain [21, 22]. Although lentiviruses can accommodate larger-size genes, they have a propensity to integrate into the host genome and exhibit limited spread throughout target brain tissue [24], thereby reducing the number of transduced cells. Given these limitations, AAVs have become widely used for gene delivery in current neuroscience research owing to their high gene transfer efficiency and low probability of integration into the host genome [25]. AAVs are also considered a safer option for gene therapy primarily because of their low immunogenicity. Furthermore, recent advancements in AAV capsid engineering have provided techniques for targeted expression in specific tissues and cell types through systemic delivery of the virus [26].

To capitalize on the potential advantages offered by AAVs and expand the versatility of monSTIM1, we here present AAV-compatible monSTIM1 variants. By implementing several strategies, we reduced the size of the monSTIM1 coding sequence, ensuring compatibility with the packaging capacity of AAVs. We demonstrate that monSTIM1 variants efficiently increase intracellular Ca^2+^ levels in diverse brain cell types, including neurons, astrocytes, and microglia. Importantly, these monSTIM1 variants retain the ultra-high light sensitivity characteristic of the original monSTIM1, enabling them to effectively drive Ca^2+^-mediated gene expression in neurons and astrocytes in the mouse brain in response to non-invasive light illumination.

## Results

### Reducing the size of monSTIM1 by replacing GFP with a small tag

The original version of monSTIM1 consists of three components: green fluorescent protein (GFP), CRY2(E281A, A9) (hereafter, CRY2), and the cytosolic STIM1 fragment (a.a. 238–685) (Fig. 1B). To reduce the coding sequence size of monSTIM1, we initially replaced the GFP of monSTIM1 with a smaller labeling component (HA or FLAG tag) and expressed each variant together with the red fluorescence Ca^2+^ indicator, R-GECO1 [27], in HeLa cells. Upon blue light stimulation, FLAG-monSTIM1 promoted Ca^2+^ influx with an efficiency comparable to that of the original GFP-monSTIM1, whereas HA-monSTIM1 exhibited a diminished Ca^2+^ increase compared with the original (Fig. 1C–E). Interestingly, HA-tagged monSTIM1 exhibited slower activation and faster deactivation kinetics than GFP- or FLAG-tagged monSTIM1 (Fig. 1F, G), indicating that the properties of monSTIM1 might be affected to some degree by labeling components. As expected, a negative control construct—FLAG-monSTIM1 conjugated with the light-insensitive CRY2(D387A) mutant—did not cause an increase in Ca^2+^ levels. Both FLAG- and HA-monSTIM1 variants conjugated with CRY2 showed similar sensitivities to blue light (Fig. 1H), indicating that light sensitivity is primarily determined by the CRY2 photoreceptor. By comparison, OptoCRAC [19], a LOV2-based method, showed diminished light sensitivity compared with monSTIM1 variants and induced a significantly smaller increase in Ca^2+^ (Fig. 1C–E, H), consistent with a previous report [16]. These results indicate that replacement of GFP with a small tag significantly reduced the size of monSTIM1 while maintaining its functionality.

### Reducing the size of monSTIM1 by truncating STIM1

**Fig. 2:**
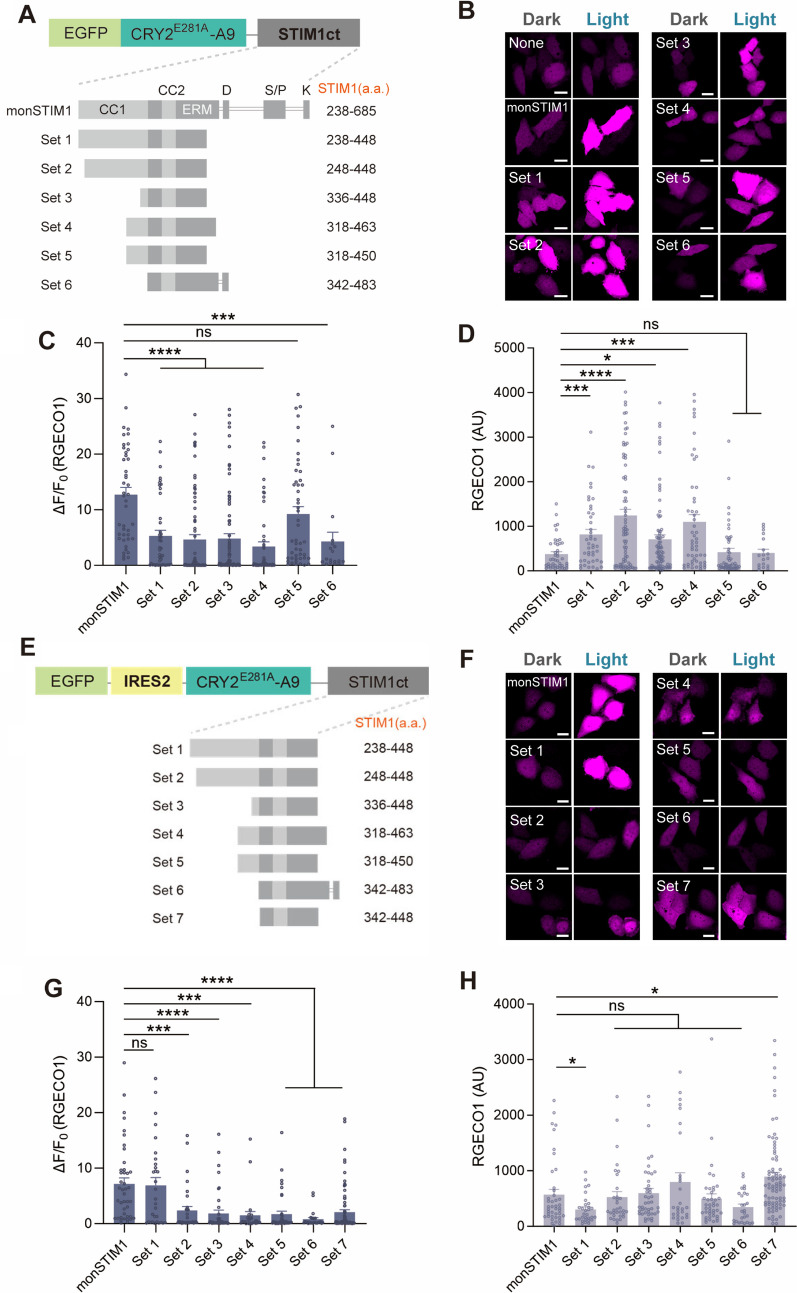
Reducing the size of monSTIM1 by truncating STIM1. **A** Schematic depiction of CRY2-fused STIM1 fragment constructs. Each fusion protein was labeled with EGFP. CC1 and CC2, coiled-coil domains; ERM, ezrin-radixin-moesin domain; D, acidic region; S/P, serine-/proline-rich segment; K, polybasic domain. **B** Fluorescence images of RGECO1 in HeLa cells co-expressing each CRY2-fused STIM1 fragment (Sets 1–6), illuminated with blue light. **C** Summary data showing maximum fold changes (ΔF/F_0_) in RGECO1 intensity upon activation of monSTIM1 variants. **D** Fluorescence intensity of RGECO1 in the absence of blue light. monSTIM1: n = 43; Set 1: n = 44; Set 2: n = 74; Set 3: n = 81; Set 4: n = 52; Set 5: n = 48; Set 6: n = 18 cells. **E** Schematic depiction of CRY2-fused STIM1 fragment constructs whose expression is driven by IRES2. **F** Fluorescence images of RGECO1 in HeLa cells co-expressing each CRY2-fused STIM1 fragment (Sets 1–7), illuminated with blue light. **G** Summary data showing maximum fold changes (ΔF/F_0_) in RGECO1 intensity upon activation of monSTIM1 variants. **H** Fluorescence intensity of RGECO1 in the absence of blue light. monSTIM1: n = 42; Set 1: n = 30; Set 2: n = 32; Set 3: n = 43; Set 4: n = 27; Set 5: n = 41; Set 6: n = 26; Set 7: n = 81 cells. Data are presented as means ± SEM (*p < 0.05, ***p < 0.001, ****p < 0.0001; Student two-tailed *t* test); ns, not significant (*p* > 0.05)

Next, we generated a series of fusion proteins to test whether truncation of STIM1 could further reduce the size of monSTIM1 while retaining the efficiency of its light-induced Ca^2+^ influx and minimal basal Ca^2+^ levels in the dark. We designed six fusion proteins of CRY2-fused STIM1 fragments encompassing the STIM1 CRAC-activation domain (CAD; a.a. 342–448) [28], which is responsible for binding to and opening Ca^2+^ channels (Fig. 2A). Each truncated STIM1 construct was flanked by different domain regions responsible for autoinhibition of CAD. We found that there was no significant difference in the averaged levels of expressed CRY2-fused STIM1 proteins (Additional file 1: Fig. S1A-B). Of the six constructs, STIM1 construct of Set 5 (a.a. 318–450–fused EGFP-CRY2) exhibited levels of Ca^2+^ influx upon light stimulation and basal R-GECO1 intensities in the dark similar to those of monSTIM1 (Fig. 2B–D). Although Sets 1–4 also induced Ca^2+^ influx, they showed elevated intensities of R-GECO1 fluorescence in the dark state, indicating an increase in basal Ca^2+^ level, possibly due to distinct levels of autoinhibition in STIM1 domains. Notably, basal R-GECO1 intensity in each cell was positively correlated with the expression level of EGFP-CRY2-STIM1, implying that a subpopulation of CRY2-STIM1 molecules is active even in the absence of blue light (Additional file 1: Fig. S1C).

To determine whether the basal intensity of R-GECO1 could be decreased by reducing the expression level of CRY2-STIM1 variants, we employed an IRES2 (internal ribosome entry sequence 2) to drive protein expression (Fig. 2E). This maneuver produced a noticeable decrease in basal R-GECO1 fluorescence, especially in Set 1 construct, which showed an even lower level of basal R-GECO1 fluorescence than monSTIM1 (Fig. 2F–H). However, Set 7 exhibited a significantly higher basal R-GECO1 intensity due to the inclusion of the constitutively active domain of STIM1 [28]. Notably, unlike in Fig. 2C, Set 5 construct expressed by IRES2 did not efficiently lead to Ca^2+^ influx under light stimulation. Only Set 1 construct was able to induce a Ca^2+^ increase at a comparable level to monSTIM1, suggesting that the expression levels of CRY2-fused STIM1 proteins can influence both the basal Ca^2+^ level in the dark and the maximal Ca^2+^ level under light illumination.

### Splitting monSTIM1 into two components

**Fig. 3:**
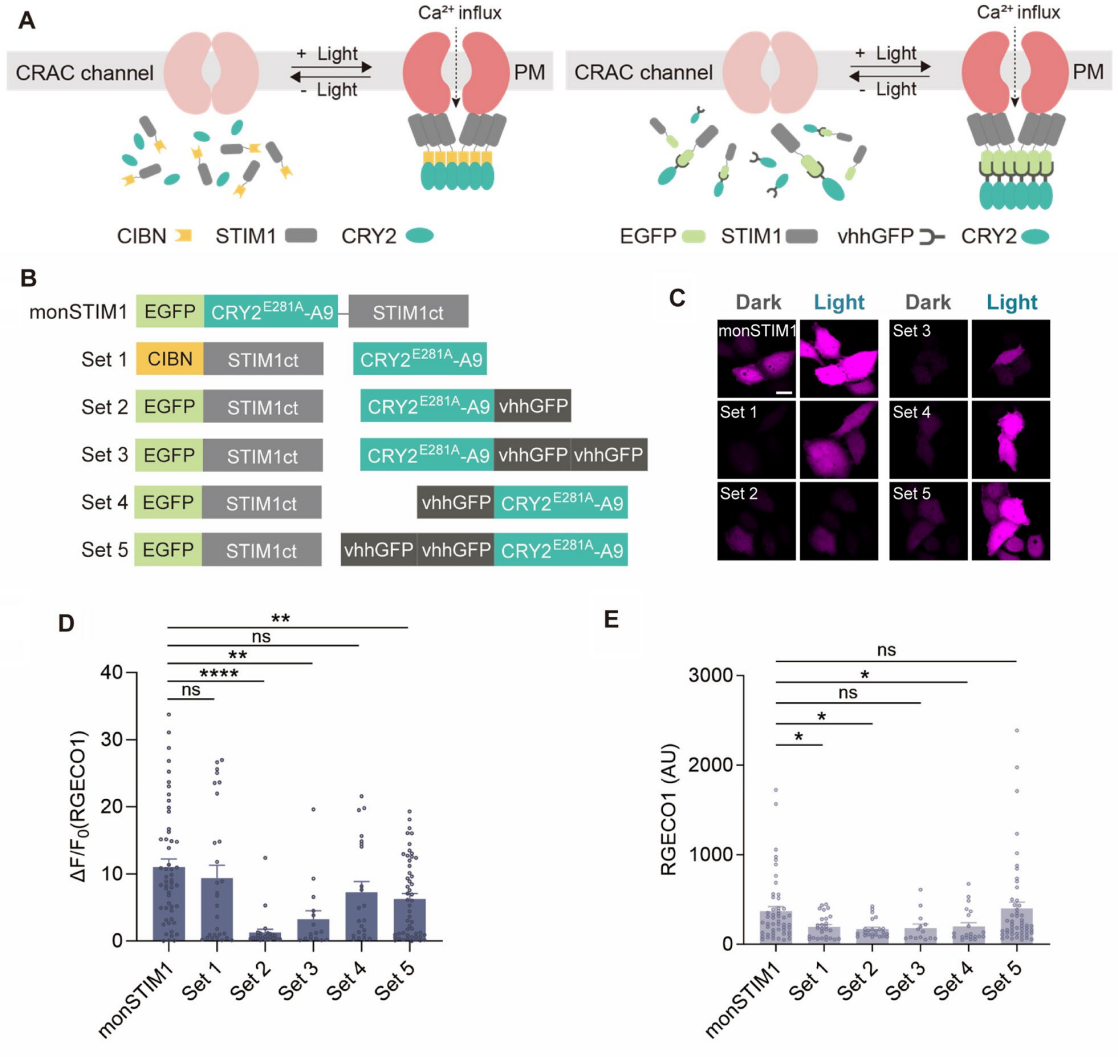
Splitting monSTIM1 into two components.** A** Schematic of the working mechanism of two-component systems. **B** Schematic of fusion constructs. **C** Fluorescence images of RGECO1 in HeLa cells co-expressing each pair of proteins (Sets 1–5), illuminated with blue light. **D** Summary data showing maximum fold changes (ΔF/F0) in RGECO1 intensity upon activation of monSTIM1 variants. **E** Fluorescence intensity of RGECO1 in the absence of blue light. monSTIM1: n = 52; Set 1: n = 27; Set 2: n = 24; Set 3: n = 14; Set 4: n = 21; Set 5: n = 51 cells. Data are presented as means ± SEM (*p < 0.05, **p < 0.01, ****p < 0.0001; Student two-tailed *t* test); ns, not significant (*p* > 0.05)

Next, we tested two approaches for splitting monSTIM1 protein into two components, ensuring that each was within the packaging limit of AAVs (Fig. 3A, B). In the first strategy (Set 1), we conjugated STIM1(238–685) to CIBN, an N-terminal fragment of the CIB1 (cryptochrome-interacting basic-helix-loop-helix 1) protein, which binds to oligomerized CRY2 in a light-dependent manner [29]. In the second strategy (Sets 2–5), we utilized GFP-labeled STIM1 and GFP nanobody (vhhGFP)-conjugated CRY2. The GFP nanobody is a single-domain antibody derived from a variable domain of a heavy-chain antibody and can specifically bind to GFP with high affinity (*K*_d_ = 0.23 nM) [30]. Therefore, the GFP-vhhGFP interaction would efficiently transmit the homo-interactions of CRY2 necessary for STIM1 oligomerization. To determine the optimal configuration of fusion proteins and copy number of vhhGFP, we fused one or two copies of vhhGFP to either the N- or C-terminus of CRY2 (Fig. 3B). Upon blue light stimulation of cells co-expressing each pair of proteins together with R-GECO1, Set 1 exhibited the greatest increase in R-GECO1 fluorescence (Fig. 3C, D). Additionally, N-terminal fusion of vhhGFP to CRY2 (Sets 4 and 5) resulted in effective induction of Ca^2+^ influx, indicating that the N-terminal fusion of vhhGFP to CRY2 retained the property of GFP binding and light-dependent oligomerization of CRY2. In contrast, C-terminal fusion proteins (Sets 2 and 3) showed significantly lower levels of Ca^2+^ increase, suggesting the C-terminal fusion interfered with GFP binding and/or light-dependent oligomerization of CRY2. Notably, none of the candidates exhibited an increase in basal R-GECO1 fluorescence in the dark, indicating that the STIM1 fragment (a.a. 238–685) sufficiently inhibited CAD activity in the absence of light stimulation (Fig. 3E). Taken together, these results identify five monSTIM1 variants that are potentially compatible with AAVs: FLAG-CRY2-STIM1(238–685) (FLAG-monSTIM1), GFP-CRY2-STIM1(318–450), IRES2-GFP-CRY2- STIM1(238–448), CIBN-STIM1(238–685) + CRY2, and GFP-STIM1(238–685) + vhhGFP-CRY2 (Additional file 2: Movie S1). Information on the size of the coding sequence for each variant is summarized in Table 1.

**Table 1:** Sizes of DNA coding sequences for mammalian expression vectors and DNA sequences between ITRs in AAV vectors

Mammalian expression vectors	Size (bp)
Single component	
EGFP-CRY2-STIM1(238–685)	3555
HA-CRY2-STIM1(238–685)	2817
FLAG-CRY2-STIM1(238–685)	2868
EGFP-CRY2-STIM1(318–450)	2610
EGFP-IRES2-CRY2-STIM1(238-448)	3432
Two components	
vhhGFP-CRY2	1920
EGFP-CRY2(238–685)	2061
CIBN-STIM1(238–685)	1854
CRY2	1494

AAV expression vectors	Size (bp) spanning ITRs
AAV-CaMKIIα-FLAG-STIM1(238–685)	4147
AAV-CaMKIIα-EGFP-CRY2-STIM1(318–450)	3864
AAV-GfaABC1D-FLAG-STIM1(238–685)	4467

### Measurement of relative Ca^2+^ levels by Fura-2 imaging

In the results described above, we utilized R-GECO1 signals as a surrogate measurement of relative Ca^2+^ levels. To obtain a more precise analysis of Ca^2+^ levels in both dark and light conditions, we performed Fura-2 imaging on cells expressing each monSTIM1 variant (Additional file 1: Fig. S2). We verified that all proteins induced significant increases in Ca^2+^ concentration upon light stimulation. In the absence of blue light, all variants, except GFP-CRY2-STIM1(318–450), exhibited comparable levels of basal Ca^2+^ to those observed in non-transfected cells, consistent with the trends observed using R-GECO1. The slightly elevated Ca^2+^ level observed in GFP-CRY2-STIM1(318–450) suggests that the N-terminal autoinhibitory domain of STIM1 was insufficient to completely suppress the activity of CAD.

### Light-induced Ca^2+^ influx in neurons and glial cells

**Fig. 4:**
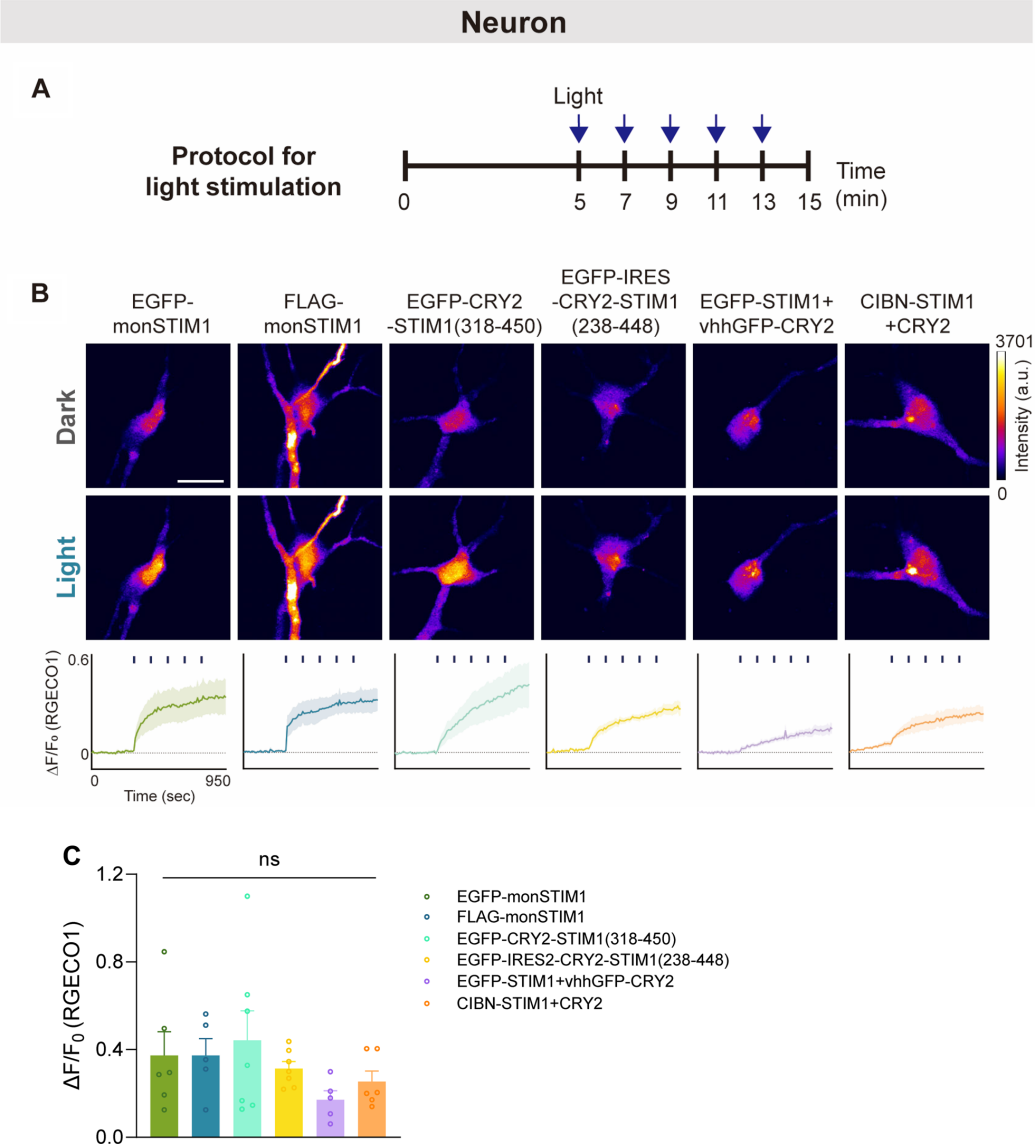
Increasing Ca^2+^ in cultured hippocampal neurons through activation of monSTIM1 variants. **A** Schematic illustrating a protocol for light illumination. **B** Fluorescence images of RGECO1 in neurons co-expressing each monSTIM1 variant, illuminated with blue light (top). Summary data showing relative changes in RGECO1 intensity over time upon repeated light illumination (bottom). **C** Summary data showing maximum fold changes (ΔF/F0) in RGECO1 intensity upon activation of monSTIM1 variants. EGFP-monSTIM1: n = 6; FLAG-monSTIM1: n = 5; EGFP-CRY2-STIM1(318–450): n = 7; EGFP-IRES2-CRY2-STIM1(238–448): n = 7; EGFP-STIM1 + vhhGFP-CRY2: n = 5; CIBN-STIM1 + CRY2: n = 6 cells. Data are presented as means ± SEM (one-way ANOVA followed by multiple comparison test); ns, not significant (*p* > 0.05)

**Fig. 5:**
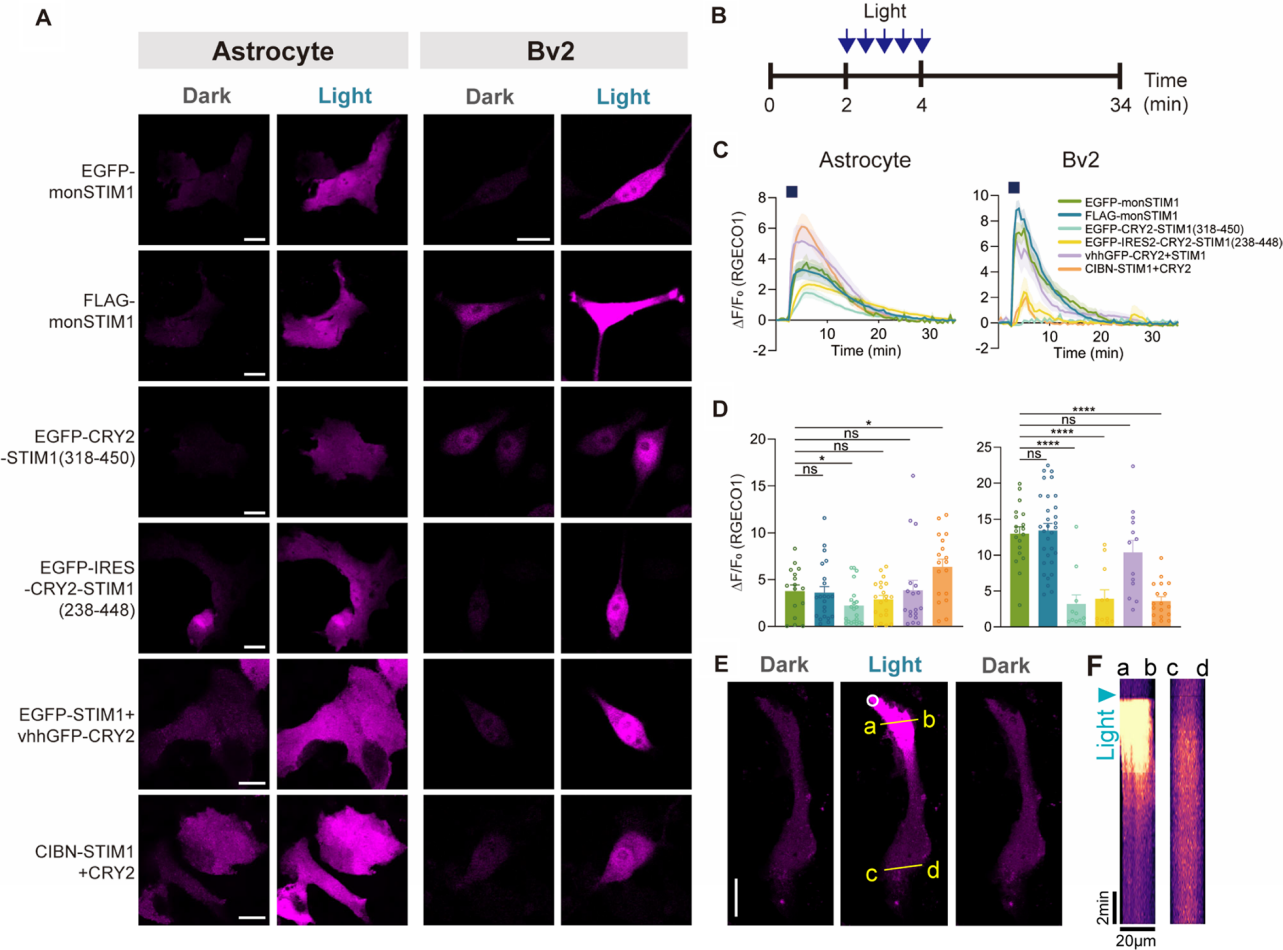
Increasing Ca^2+^ in cultured astrocytes and microglia through activation of monSTIM1 variants. **A** Fluorescence images of RGECO1 in astrocytes (left) and microglia (BV2 cells, right) co-expressing each monSTIM1 variant, illuminated with blue light. **B** Schematic illustrating the protocol for light illumination. **C** Summary data showing relative changes in RGECO1 intensity over time upon light illumination. **D** Summary data showing maximum fold changes (ΔF/F0) in RGECO1 intensity upon activation of monSTIM1 variants. EGFP-monSTIM1: n = 16; FLAG-monSTIM1: n = 22; EGFP-CRY2-STIM1(318–450): n = 21; EGFP-IRES2-CRY2-STIM1(238–448): n = 21; EGFP-STIM1 + vhhGFP-CRY2: n = 18; CIBN-STIM1 + CRY2: n = 18 cells (astrocyte). EGFP-monSTIM1: n = 19; FLAG-monSTIM1: n = 31; EGFP-CRY2-STIM1(318–450): n = 11; EGFP-IRES2-CRY2-STIM1(238–448): n = 11; EGFP-STIM1 + vhhGFP-CRY2: n = 13; CIBN-STIM1 + CRY2: n = 18 cells (BV2). **E** Fluorescence images of RGECO1 in astrocytes upon light illumination of a subcellular region indicated by a white-lined circle. **F** Kymograph of RGECO1 corresponding to lines a-b and c-d in (**E**). Data are presented as means ± SEM (*p < 0.05, ****p < 0.0001; Student two-tailed *t* test); ns, not significant (*p* > 0.05)

To access the efficacy of monSTIM1 variants in modulating Ca^2+^ signals in the brain, we introduced these proteins into cultured neurons, astrocytes, and microglia. In neurons expressing monSTIM1 variants, we observed rapid and sustained increases in intracellular Ca^2+^ levels upon repeated light exposure (five times at 2-min intervals) (Fig. 4A, B and Additional file 3: Movie S2). There was no significant difference in the levels of Ca^2+^ increase across monSTIM1 variants (Fig. 4C). Interestingly, despite variations in maximum fold changes among the different variants, Ca^2+^ levels were remarkably elevated in astrocytes and microglia for all variants upon light stimulation (Fig. 5A–D, Additional file 4: Movie S3 and Additional file 5: Movie S4). Notably, the extent of the increase in Ca^2+^ signals induced in glial cells by monSTIM1 variants surpassed that observed in neurons, an effect that could be attributable to variations in the expression levels or densities of endogenous Ca^2+^ channels across different cell types. Next, to investigate the local control of Ca^2+^ levels by monSTIM1, we expressed FLAG-monSTIM1 in astrocytes and applied light for 1 s to a specific subcellular region. Remarkably, this focal stimulus produced a reversible and localized increase in Ca^2+^ levels within the illuminated area, without causing a detectable increase in Ca^2+^ signals in the opposite subcellular region (Fig. 5E, F). These findings demonstrate the capability of monSTIM1 variants to effectively modulate intracellular Ca^2+^ levels in neurons and glial cells in both space and time.

### Application of monSTIM1 variants in vivo in the mouse brain

**Fig. 6:**
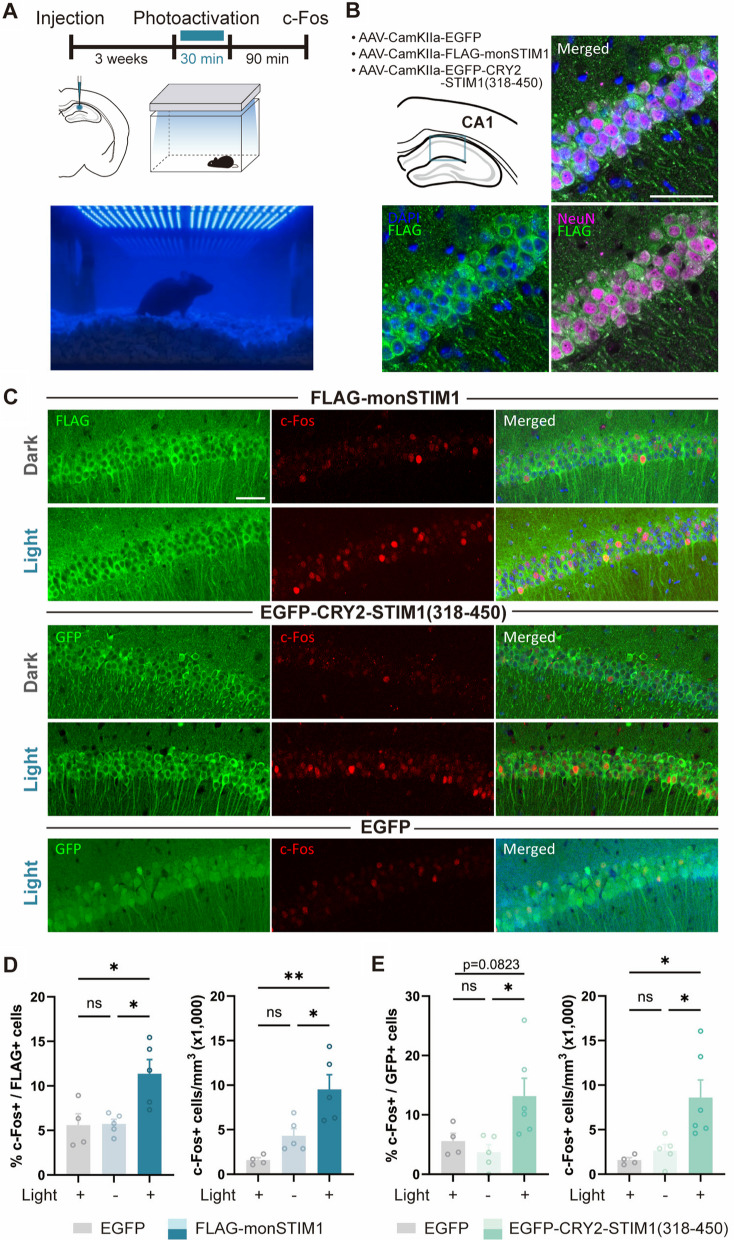
Application of monSTIM1 variants in neurons.** A** Top: Schematic of the experiment. AAV-compatible monSTIM1 variants are expressed under the control of a CaMKIIα promoter targeted to the hippocampal CA1 region. Bottom: Mice were illuminated with blue LED light via a customized transcranial light illumination system and perfused for immunohistochemistry. **B** Schematic displaying the areas used for the quantification of active neurons expressing cFos and monSTIM1 variants in the CA1 region. Two AAV-compatible monSTIM1 variants—AAV-CaMKIIα-FLAG-monSTIM1 and AAV-CaMKIIα-EGFP-STIM1(318–450)—were used. Representative images showing CA1 neurons expressing FLAG-monSTIM1. Blue, DAPI; Green, FLAG; Magenta, NeuN. Scale bar, 50 μm. **C** Representative images showing cFos-positive cells expressing each monSTIM1 variants, with or without non-invasive light delivery, and EGFP (control). Scale bar, 50 μm. **D** Quantification of cFos-positive cells expressing FLAG-monSTIM1. **E** Quantification of cFos-positive cells expressing EGFP-STIM1(318–450). EGFP: n = 4; FLAG-monSTIM1 (-Light): n = 5; FLAG-monSTIM1 (+ Light): n = 5; EGFP-CRY2-STIM1(318–450) (-Light): n = 5; EGFP-CRY2-STIM1(318–450) (+ Light): n = 6 mice. Data are presented as means ± SEM (*p < 0.05, **p < 0.01; one-way ANOVA followed by Holm-sidak’s multiple comparison test); ns, not significant (*p* > 0.05)

Next, we investigated whether monSTIM1 variants could modulate Ca^2+^ signaling in vivo in the mouse brain. To this end, we generated AAV vectors using two single-component variants—FLAG-monSTIM1 and GFP-CRY2-STIM1(318–450). To further increase the available space for packaging the coding sequence, we additionally replaced WPRE and hGH polyadenylation signal in the AAV cassette with W3SL, a smaller component that enhances the expression of a transgene [31]. We utilized the small CaMKIIα promoter [32] to selectively express monSTIM1 variants in excitatory neurons. Three weeks after virus injection into the hippocampal CA1 region (Fig. 6A), monSTIM1 variants showed widespread and selective expression in neurons (NeuN-positive cells) (Fig. 6B). Blue light-mediated activation of monSTIM1 variants led to a significant induction of cFos, a Ca^2+^-responsive immediate-early gene, in approximately 10% of STIM1-positive neurons (Fig. 6C–E). In contrast, mice under dark conditions (ambient light) and those expressing GFP alone under light-stimulation conditions did not exhibit detectable increases in cFos expression, indicating that cFos expression was specifically driven by the activity of monSTIM1 variants and not by light illumination per se. Importantly, it should be noted that expression of the gene was successfully induced by non-invasive light illumination, indicating that these two variants (FLAG-monSTIM1 and GFP-CRY2-STIM1(318–450)) still possessed ultra-high light sensitivity similar to the original version of monSTIM1 [22].

**Fig. 7:**
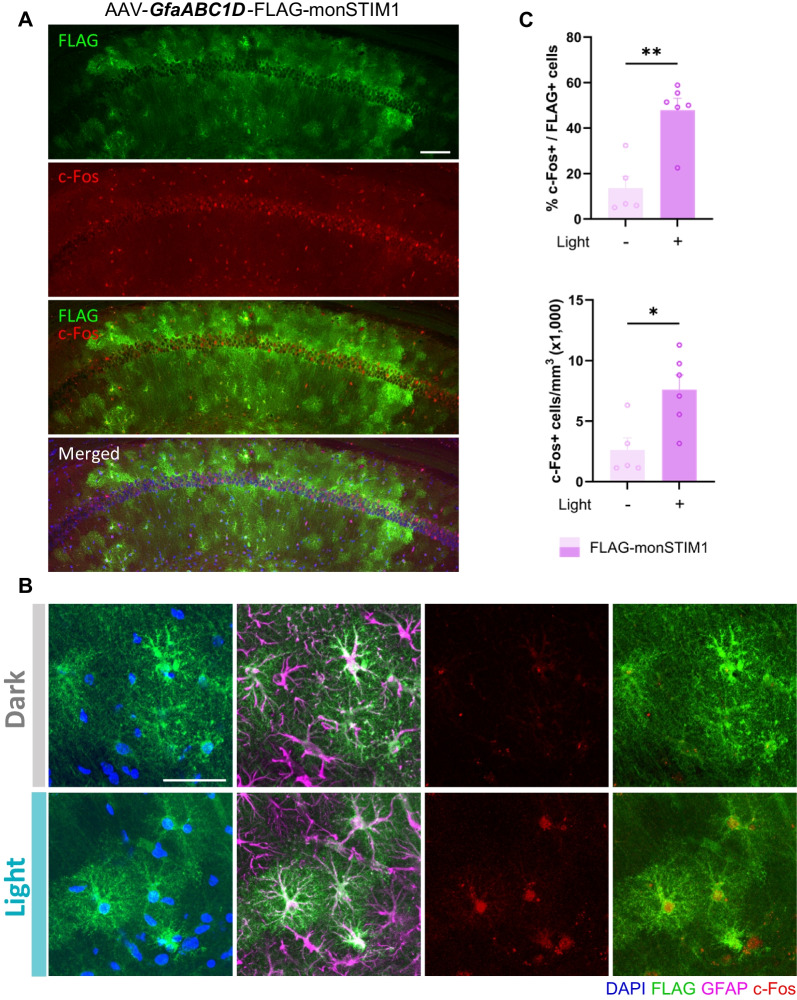
Application of monSTIM1 variants in astrocytes. **A** Representative images showing FLAG-monSTIM1 expressed under the control of a GfaABC1D promoter targeted to the hippocampal CA1 region. Green, FLAG; Red, c-Fos; Blue, DAPI. Scale bar, 100 μm. **B** Representative images showing cFos-positive cells expressing FLAG-monSTIM1, with or without non-invasive light delivery. Green, FLAG; Blue, DAPI; Magenta, GFAP; Red, c-Fos. Scale bar, 50 μm. **C** Quantification of cFos-positive cells expressing FLAG-monSTIM1 in CA1 astrocytes. FLAG-monSTIM1 (-Light): n = 5; FLAG-monSTIM1 (+ Light): n = 6 mice. Data are presented as means ± SEM (*p < 0.05, **p < 0.01; Unpaired student’s *t* test); ns, not significant (*p* > 0.05)

To evaluate the ability of monSTIM1 to modulate Ca^2+^ signaling in astrocytes, we expressed FLAG-monSTIM1, driven by the GfaABC1D promoter, in CA1 astrocytes [33] and confirmed its selective expression in GFAP-positive cells (Fig. 7A, B). Upon light stimulation, cFos expression was robustly increased in approximately 50% of monSTIM1-positive astrocytes, whereas monSTIM1-negative astrocytes showed no such response (Fig. 7B, C). Taken together, these results demonstrate that non-invasive light stimulation of the examined monSTIM1 variants effectively and selectively activates Ca^2+^ signaling in neurons and glial cells.

## Discussion

Here, we have developed AAV-compatible monSTIM1 variants that enable precise and selective modulation of intracellular Ca^2+^ levels in vivo in the mouse brain. Through three molecular engineering strategies, we successfully reduced the size of the coding sequence of the original monSTIM1 to fit within the packaging constraints of the AAV. Consistent with previous findings [16], these CRY2-based monSTIM1 variants induced much greater increases in Ca^2+^ levels compared with the LOV2-based method (OptoCRAC) [19]. Importantly, by virtue of the ultra-high light sensitivity of the CRY2(E281A, A9) mutant [22], the examined variants (FLAG-monSTIM1 for neurons and astrocytes and EGFP-CRY2-STIM1(318–450) for neurons) still maintained highly efficient Ca^2+^ influx and subsequent activation of signaling downstream of Ca^2+^ influx in the brains of freely moving animals through non-invasive delivery of blue light. Therefore, these tools offer a strategy for circumventing the compounding effects associated with conventional in vivo optogenetic experiments, such as tissue damage, glial scar formation, inflammation and tissue heating, that arise from the long-term insertion of optic fibers [34–37].

We found that, with the exception of EGFP-CRY2-STIM1(318–450), none of the monSTIM1 variants induced significant increases in basal Ca^2+^ levels in HeLa cells in the absence of blue light (Fig. S2), indicating that the majority of these molecules are in an inactive state. However, it is important to note that the expression of EGFP-CRY2-STIM1(318–450) in the CA1 neurons under dark conditions did not lead to any significant increase in cFos expression (Fig. 6C, E), indicating that EGFP-CRY2-STIM1(318–450) in the absence of blue light has minimal impact on calcium downstream signaling. Additionally, our observation that basal Ca^2+^ levels are positively correlated with expression levels of the proteins suggests that tight control of the expression of monSTIM1 variants through inducible gene expression systems may be necessary to address potential issues associated with basal protein activity [38–40]. Despite their effective induction of Ca^2+^ influx, the two-component systems raise concerns regarding variations in expression levels of each component in individual cells—variability that could lead to heterogeneous responses to the same light stimulation conditions. It should be noted that the strategy of truncating STIM1 holds promise for further reducing the size of the coding sequence. This would allow utilization of larger promoters for gene expression in various cell types and support the design of bicistronic vectors for simultaneous expression of monSTIM1 variants with genetically encoded fluorescent indicators to directly investigate the relationships between Ca^2+^ signals and specific molecular activities. Thus, engineering through truncation and mutagenesis of STIM1 fragments has the potential to generate even smaller monSTIM1 variants with low basal activity in the dark, further enhancing the versatility of our platform.

A number of opsin-based optogenetic tools, such as channelrhodopsin 2 (ChR2) variants, have been developed for controlling neuronal activity [41]. While these tools exhibit a remarkable ability to control the membrane potential of excitable cells, they are non-selective cation channels, allowing the entry of sodium and potassium ions in addition to Ca^2+^ [41]. Consequently, these tools are not suitable for regulating Ca^2+^-specific events or controlling the function of non-excitable cells, such as astrocytes and microglia, in the brain. In contrast, monSTIM1 specifically induces Ca^2+^ influx in both neurons and glial cells owing to the ubiquitous expression of Ca^2+^-selective CRAC channels [10]. Therefore, our tools offer attractive options for investigating spatiotemporal aspects of Ca^2+^-mediated neuronal and glial cell functions, as well as their interactions in various contexts.

AAVs have attracted significant attention for their ability to deliver genes to treat a wide range of human diseases [23]. Recent advancements in engineering the capsid proteins of AAVs have remarkably enhanced the utility of AAVs in both clinical studies and basic research [23, 26]. By combining our methods with blood–brain-barrier–penetrating AAV variants and various transgenic mice expressing recombinases, it is possible to develop non-invasive approaches for regulating Ca^2+^ activity in specific cell types within the brain, obviating the need for brain surgery. We anticipate that our optogenetic tools for controlling Ca^2+^ signals will provide a framework for developing potential therapeutic interventions for diseases associated with dysregulated Ca^2+^ signaling.

## Methods

### Plasmid construction

Expression plasmid for R-GECO1 (Addgene plasmid #32444) was obtained from Addgene. For construction of expression plasmids for HA-monSTIM1 and FLAG-monSTIM1, the monSTIM1 sequence from GFP-monSTIM1 [22] was amplified by polymerase chain reaction (PCR) using HA-F, FLAG-F, and HA-R primers. The amplified sequence was then ligated into GFP-monSTIM1 at *Nhe*I and *Sal*I sites after excising *EGFP.* The LOV2(404–546) sequence in OptoCRAC [19] was PCR-amplified using LOV2-F and LOV2-R primers and ligated into the EGFP-C1 vector at *Bsr*GI and *Hin*dIII sites to generate the EGFP-LOV2 vector. The STIM1(336–486) sequence in GFP-monSTIM1 was PCR-amplified using STIM1(336–486)-F and STIM1(336–486)-R primers and ligated into the EGFP-LOV2 vector at *Hin*dIII and *Bam*HI sites to generate the OptoCRAC expression plasmid. For construction of GFP-CRY2-STIM1(238–448), GFP-CRY2-STIM1(248–448), GFP-CRY2-STIM1(336–448) and GFP-CRY2-STIM1(342–448) expression plasmids, sequences encoding STIM1(238–448), STIM1(248–448), STIM1(336–448) and STIM1(342–448) fragments were PCR-amplified using STIM1(238–448)-F, STIM1(248–448)-F, STIM1(336–448)-F, STIM1(342–448)-F, and STIM1(238–448)-R primers and ligated into monSTIM1 at *Bsp*EI and *Bam*HI sites. For construction of GFP-CRY2-STIM1(318–463) and GFP-CRY2-STIM1(318–450) expression plasmids, sequences encoding STIM1(318–463) and STIM1(318–450) fragments were PCR-amplified using STIM1(318–463)-F, STIM1(318–463)-R and STIM1(318–450)-R primers and ligated into monSTIM1 at *Bsp*EI and *Bam*HI sites. For generation of the IRES2-CRY2-STIM1(238–448) expression plasmid, the IRES2 sequence was PCR-amplified using IRES2-F and IRES2-R primers and ligated into GFP-CRY2-STIM1(238–448) at *Nhe*I and *Bsr*GI sites after excising *EGFP*. The sequence encoding EGFP was then inserted at *Nhe*I and *Not*I sites to generate the EGFP-IRES2-CRY2-STIM1(238–448) expression plasmid. The STIM1(238–448) fragment was replaced with other STIM1 variants at *Bsp*EI and *Bam*HI sites. The CRY2(E281A, A9) sequence in monSTIM1 was PCR-amplified using CRY2-F and CRY2-R primers and ligated into EGFP-N1 at *Age*I and *Bsr*GI sites after excising *EGFP* to generate the CRY2-N1 vector. The vhhGFP sequence in mCherry-CRY2-vhhGFP [42] was PCR-amplified using vhhGFP-F1 and vhhGFP-R1 primers and ligated into CRY2-N1 at *Nhe*I and *Age*I sites to generate the vhhGFP-CRY2 expression plasmid. The CRY2(E281A, A9) sequence from CRY2-N1 was digested with *Age*I and *Bsr*GI and ligated into EGFP-C1 (Clontech) after excision of *EGFP* to generate the CRY2-C1 vector. The vhhGFP sequence in mCherry-CRY2-vhhGFP was PCR-amplified using vhhGFP-F2 and vhhGFP-R2 primers and ligated into CRY2-C1 at *Bsr*GI and *Xho*I sites to generate the CRY2-vhhGFP vector. For construction of virus vector for pAAV-CamKIIa-GFP-monSTIM1, an exchange enzyme site was designed by using *Nhe*I-*Bam*HI oligomer and inserted into pAAV-CamKIIa-EGFP (addgene #50469). GFP-monSTIM1 was ligated into pAAV-CamKIIa-EGFP at the *Nhe*I and *Bam*HI sites after excising *EGFP.* The W3SL sequence in pAAV-CW3SL-EGFP (Addgene plasmid #61463) was PCR-amplified using W3SL-F and W3SL-R primers and then ligated into pAAV-CamKIIa-GFP-monSTIM1 vector at the *Bam*HI and *Rsr*II sites after the excision of WPRE and hGH poly(A) signal to generate the pAAV-CamKIIa-GFP-monSTIM1-W3SL vector. For construction of virus vectors of pAAV-CamKIIa-FLAG-monSTIM1-W3SL and pAAV-CamKIIa-GFP-STIM1(318–450)-W3SL, FLAG-monSTIM1 or GFP-STIM1(318–450) was inserted into the pAAV-CamKIIa-GFP-monSTIM1-W3SL vector using *Nhe*I and *Bam*HI sites after excising *GFP-monSTIM1*. The GfaABC1D promoter sequence was PCR-amplified using GfaABC1D-F and GfaABC1D-R primers and ligated into pAAV-CaMKIIα-FLAG-monSTIM1 at MluI and XbaI sites after excising *CaMKIIα* promoter sequence to generate pAAV-GfaABC1D-FLAG-monSTIM1. Oligonucleotides used in this research are listed in Additional file 1: Table S1.

### Cell culture and transfection

HeLa cells (ATCC) and BV2 cells (ATCC) were maintained in Dulbecco’s Modified Eagle’s Medium (DMEM; Gibco, Cat# 11965092, MA, USA) supplemented with 10% fetal bovine serum (FBS; Gibco, Cat# 16,000–044) at 37 °C in a humidified 5% CO_2_ atmosphere. Hippocampal neurons were prepared from embryonic day 15–16 mice, and the hippocampi from collected embryos were dissected in Hank’s balanced salt solution (HBSS; Gibco, Cat# 14175-095). The collected hippocampus was dissociated by incubating with 0.05% trypsin for 5 min at 37 °C, filtered through a 0.4-μm filter, and then seeded onto a 24-well polymer-coverslip–bottom plate (ibiTreat; ibidi, Cat# 82426, Gräfelfing, Germany) coated with 50 μg/mL poly-D-lysine (Millipore, Cat# A003-E, MA, USA). Neurons were grown in Neurobasal medium Cat# 21103-049) supplemented with 2% B-27, 2% N-2 supplements, 2 mM GlutaMAX (Gibco, Cat# 35050061), and 1000 units/mL penicillin–streptomycin and maintained at 37 °C in a humidified 5% CO_2_ atmosphere. Primary cortical astrocytes were dissected by removing the adherent meninges from P0-P1 C57BL/6 mouse pups, followed by dissociation into a single-cell suspension by trituration through a Pasteur pipette. Dissociated cells were plated on a 60-mm dish coated with 50 μg/mL poly-D-lysine. Cells were grown in high-glucose DMEM (Gibco) containing L-glutamine, supplemented with 10% horse serum, 10% FBS and 1000 units/mL penicillin–streptomycin, and maintained at 37 °C in a humidified 5% CO_2_ atmosphere. Cells were transfected using Lipofectamine LTX (Invitrogen, Cat# 15338-100, CA, USA) according to the manufacturer’s instructions.

### Live-cell imaging

For live-cell imaging, a Nikon A1R confocal microscope (Nikon Instruments), mounted onto a Nikon Eclipse Ti body and equipped with a CFI Plan Apochromat VC objective (× 60/1.4-numerical aperture (NA)) and digital zoom Nikon imaging software (NIS Element AR 64-bit version 3.21; Laboratory Imaging), was used. Cells were maintained at 5% CO_2_ and 37 °C during imaging with the microscope-mounted Chamlide TC System (Live Cell Instruments, Inc., Korea). Ca^2+^ influx in cells co-expressing R-GECO1 was measured using genetically encoded red Ca^2+^ indicators with each of the monSTIM1 variants; 488 and 561 nm laser sources were used to excite cells at 30-s intervals. Captured images were analyzed using NIS-Elements AR microscope imaging software (NIS-element AR 64-bit version 3.21; Nikon). Time-lapse images of Ca^2+^ influx were analyzed in defined regions of interest (ROI) in the cells, and changes in R-GECO1 intensity were quantified.

### Fura-2 imaging

HeLa cells were loaded with Fura-2 AM (Invitrogen, Cat# F6774), dissolved in dimethyl sulfoxide and diluted to 2 μM in DMEM, by incubating at room temperature for 30 min. The cells were then washed three times for 5 min at each step. Fura-2 imaging was performed using a LAMBDA DG-4 lamp (Sutter Instrument Company) and a × 40/0.75 NA CFI Plan Fluor objective, with intermittent excitation using 340 and 380 nm filtered fluorescent light. The emitted light was collected with a Nikon DS-Qi1 monochrome digital camera after passing through a 510-nm emission filter.

### Mice

Male C57BL/6 mice, 12–15 weeks old, were group-housed on a 12-h light/dark cycle with ad libitum access to food and water. Experimental protocols were approved by the Institutional Animal Care and Use Committee of IBS (Daejeon, Korea). Mice were randomly assigned to experimental groups.

### Stereotaxic surgery and in vivo light-stimulation conditions

Surgical procedures were performed under IBS IACUC guidelines. Anesthesia in mice was induced with 5% isoflurane and maintained with 1–2% isoflurane during the stereotaxic surgery. After securing the skull in a stereotaxic apparatus (RWD), the skin was shaved and the scalp was sterilized with povidone-iodine, and a small craniotomy was performed. The following coordinates were used for microinjections into the CA1: AP, 2.0 mm, ML, ± 1.5 mm, DV, -1.4 mm. All microinjections were carried out using a glass capillary Picospritzer (Parker). Following each injection, the capillary was kept in place for an additional 10 min to allow for diffusion, and then slowly withdrawn. In vivo light-stimulation experiments were performed 3 weeks post injection using 473-nm light, delivered via a solid-state LED excitation system (Live Cell Instrument) [22]. The duration of light illumination was 30 min.

### Immunohistochemistry

Mice were anesthetized with mixture of Alfaxan (40 mg/kg) and xylazine (10 mg/kg) 90 min after dark or light-illumination conditions and transcardially perfused first with PBS and then with PBS containing 4% paraformaldehyde (PFA). Brains were extracted, postfixed overnight in 4% PFA at 4 ℃, and then sectioned to a thickness of 30 μm using a vibratome (Leica). For immunostaining, free-floating sections were washed in PBS, incubated for 1 h in blocking solution (PBS containing 5% normal goat serum and 0.3% Triton X-100) and incubated overnight at 4℃ with primary antibodies (see below) in blocking solution. Sections were then washed in PBS, incubated for 2 h at room temperature with secondary antibodies (see below) in PBS. Finally, brain tissue sections were washed in PBS and slide-mounted with Vectashield antifade mounting medium containing DAPI (4’,6-diamidino-2-phenylindole) (Vector Laboratories, Cat# H-1200, CA, USA). For CA1 imaging, fluorescence images were obtained using a Leica Stellaris 8 confocal microscope with 20×, 40×, and 60 × objectives. For all brain regions, fluorescence images were acquired using a Zeiss Axio scan Z1 with 10 × objectives. Images were analyzed using ImageJ (NIH). More than three coronal brain sections were analyzed for each mouse sample for quantification of cFos^+^ and FLAG^+^ cells.

### Antibodies

#### Primary antibodies

The following primary antibodies were used: rabbit anti-cFos (diluted 1:2000; Cell Signaling Technologies, Cat# 2250, MA, USA), rat anti-FLAG (diluted 1:500; BioLegend, Cat# 637303, CA, USA), chicken anti-GFAP (diluted 1:1000; Millipore, Cat# AB5541), chicken anti-GFP (diluted 1:1000; Invitrogen, Cat# A10262).

#### Secondary antibodies

The following secondary antibodies were used: Alexa Fluor 488-conjugated goat anti-rabbit (diluted 1:1000; Invitrogen, Cat# A11034), Alexa Fluor 594-conjugated goat anti-rat (diluted 1:1000; Invitrogen, Cat# A11007), Alexa Fluor 647-conjugated goat anti-chicken (diluted 1:1000; Invitrogen, Cat# A32933), Alexa Fluor 488-conjugated goat anti-chicken (diluted 1:1000; Invitrogen, Cat# A11039), and Alexa Fluor 594-conjugated goat anti-rabbit (diluted 1:1000; Invitrogen, Cat# A11037).

### Supplementary Information

**Additional file 1: Figure S1.** Correlation between protein expression level and basal RGECO1 intensity. **A** Schematic depiction of CRY2-fused STIM1 fragment constructs. **B** Summary data showing expression levels of monSTIM1 variants. Data presented as means ± SEM (one-way ANOVA followed by multiple comparison test); ns, not significant (*p *> 0.05). **C** Graphs showing correlation between expression level of CRY2-fused STIM1 fragment and the basal RGECO1 intensity. monSTIM1: n = 110; Set 1: n = 85; Set 2: n = 104; Set 3: n = 150; Set 4: n = 136; Set 5: n = 104; Set 6: n = 56 cells. Scattered plots were analyzed by simple linear regression. **Figure S2.** Measurement of relative Ca^2+^ levels by Fura-2 imaging in cells expressing monSTIM1 variants. **A** Fura-2 ratio (Emission 340 nm/380 nm) measured in the dark condition. **B** Fura-2 ratio measured after blue light illumination. EGFP-monSTIM1: n = 120; FLAG-monSTIM1: n = 150; EGFP-CRY2-STIM1(318–450): n = 201; EGFP-IRES2-CRY2-STIM1(238–448): n = 146; EGFP-STIM1 + vhhGFP-CRY2: n = 152; CIBN-STIM1 + CRY2: n = 144 cells. Data are presented as means ± SEM (****p < 0.0001; Student two-tailed *t* test). **Table S1.** Oligos used in this study.

**Additional file 2: Movie S1.** Reversible Ca^2+^ increase in HeLa cells expressing monSTIM1 variants by light illumination.

**Additional file 3: Movie S2.** Light-induced Ca^2+^ increase in cultured hippocampal neurons expressing monSTIM1 variants.

**Additional file 4: Movie S3.** Reversible Ca^2+^ increase in cultured astrocytes expressing monSTIM1 variants by light illumination.

**Additional file 5: Movie S4.** Reversible Ca^2+^ increase in microglia (BV2) expressing monSTIM1 variants by light illumination.

## Data Availability

The data that support the findings of this study are available from the corresponding author upon reasonable request.

## Abbreviations

AAV: Adeno-associated virus
CAD: CRAC-activation domain
CRAC: Calcium release-activated calcium
CRY2: Cryptochrome 2
ER: Endoplasmic reticulum
EGFP: Enhanced green fluorescent protein
hGH: Human growth hormone
IRES2: Internal ribosome entry sequence 2
ITR: Inverted terminal repeats
LOV2: Light-oxygen-voltage-sensing 2
GECO: Genetically encoded calcium indicator for optical imaging
STIM1: Stromal interaction molecule 1
WPRE: Woodchuck hepatitis virus post-transcriptional regulatory element
