# Supplementary material for: AAV-compatible optogenetic tools for activating endogenous calcium channels in vivo
Source: Mol Brain. 2023 Oct 17;16:73. doi: 10.1186/s13041-023-01061-7 (PMC10583393; doi:10.1186/s13041-023-01061-7)
Supplement: Supplementary file 1 — Additional file 1: Figure S1. Correlation between protein expression level and basal RGECO1 intensity. A Schematic depiction of CRY2-fused STIM1 fragment constructs. B Summary data showing expression levels of monSTIM1 variants. Data presented as means ± SEM (one-way ANOVA followed by multiple comparison test); ns, not significant (p > 0.05). C Graphs showing correlation between expression level of CRY2-fused STIM1 fragment and the basal RGECO1 intensity. monSTIM1: n = 110; Set 1: n = 85; Set 2: n = 104; Set 3: n = 150; Set 4: n = 136; Set 5: n = 104; Set 6: n = 56 cells. Scattered plots were analyzed by simple linear regression. Figure S2. Measurement of relative Ca2+ levels by Fura-2 imaging in cells expressing monSTIM1 variants. A Fura-2 ratio (Emission 340 nm/380 nm) measured in the dark condition. B Fura-2 ratio measured after blue light illumination. EGFP-monSTIM1: n = 120; FLAG-monSTIM1: n = 150; EGFP-CRY2-STIM1(318–450): n = 201; EGFP-IRES2-CRY2-STIM1(238–448): n = 146; EGFP-STIM1 + vhhGFP-CRY2: n = 152; CIBN-STIM1 + CRY2: n = 144 cells. Data are presented as means ± SEM (****p < 0.0001; Student two-tailed t test). Table S1. Oligos used in this study. [file 13041_2023_1061_MOESM1_ESM.pdf]

Additional file 1

AAV-compatible optogenetic tools for activating endogenous calcium channels in vivo

Yeon Hee Kook[1,2], Hyoin Lee[1], Jinsu Lee[3], Yeonji Jeong[3], Jaerang Rho[2], Won Do Heo[3,4*], Sangkyu Lee[1*]

[1]Center for Cognition and Sociality, Institute for Basic Science (IBS), Daejeon 34126, Republic of Korea

[2]Department of Bioscience and Biotechnology, Graduate School, Chungnam National University, Daejeon 34134, Korea

[3]Department of Biological Sciences, Korea Advanced Institute of Science and Technology (KAIST), Daejeon 34141, Republic of Korea

[4]KAIST Institute for the BioCentury, Korea Advanced Institute of Science and Technology (KAIST), Daejeon 34141, Republic of Korea

These authors contributed equally: Yeon Hee Kook, Hyoin Lee, Jinsu Lee

*Correspondence: Won Do Heo wdheo@kaist.ac.kr, Sangkyu Lee sklee@ibs.re.kr

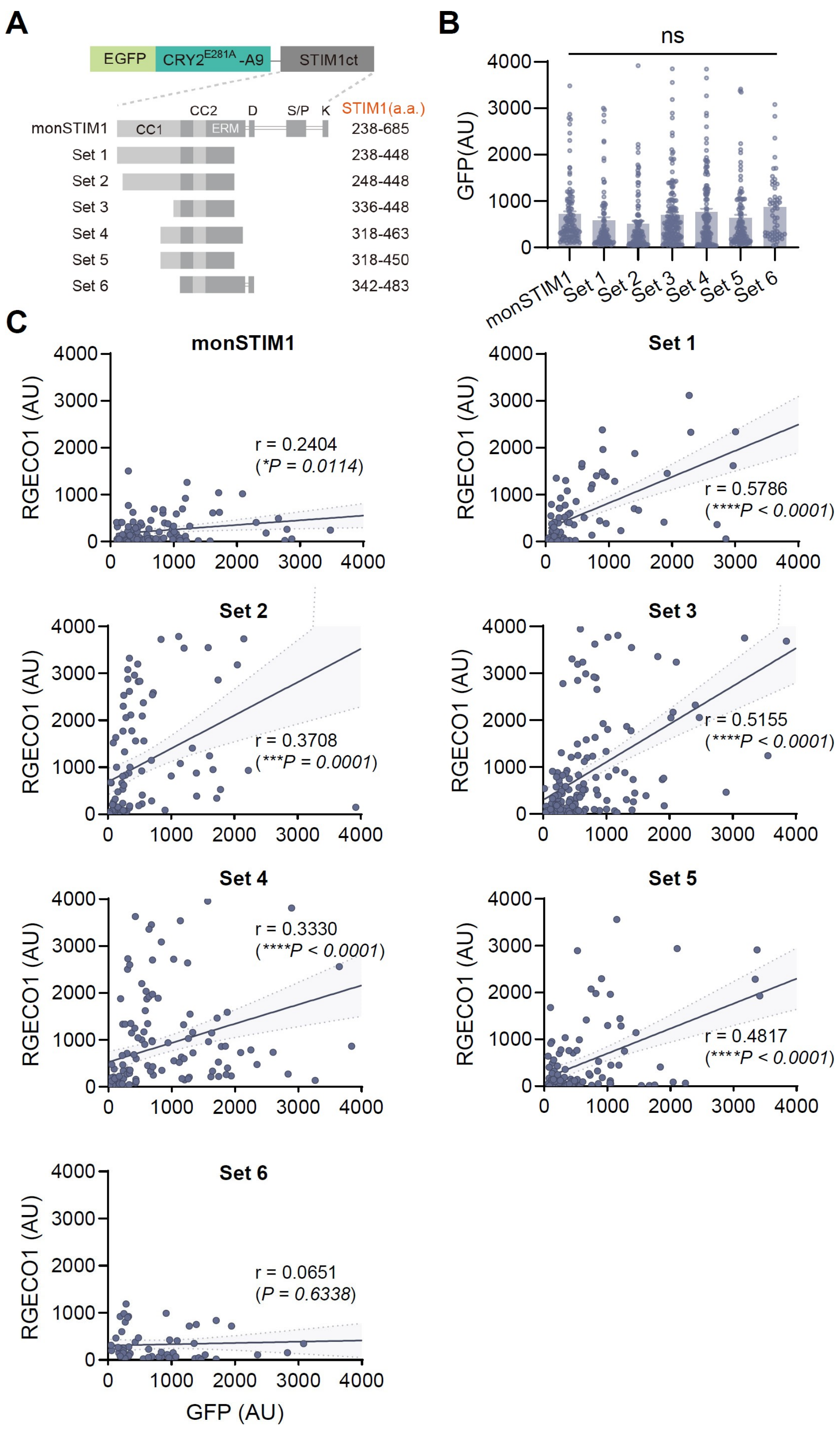

Figure S1. Correlation between protein expression level and basal RGECO1 intensity.

(A) Schematic depiction of CRY2-fused STIM1 fragment constructs. **(B)** Summary data showing expression levels of monSTIM1 variants. Data presented as means ± SEM (one-way ANOVA followed by multiple comparison test); ns, not significant (*p*>0.05) **(C)** Summary data showing the correlation between expression levels of the CRY2-fused STIM1 fragment and basal RGECO1 intensity. monSTIM1: n=110; Set 1: n=85; Set 2: n=104; Set 3: n=150; Set 4: n=136; Set 5: n=104; Set 6: n=56 cells. Scattered plots were analyzed by simple linear regression.

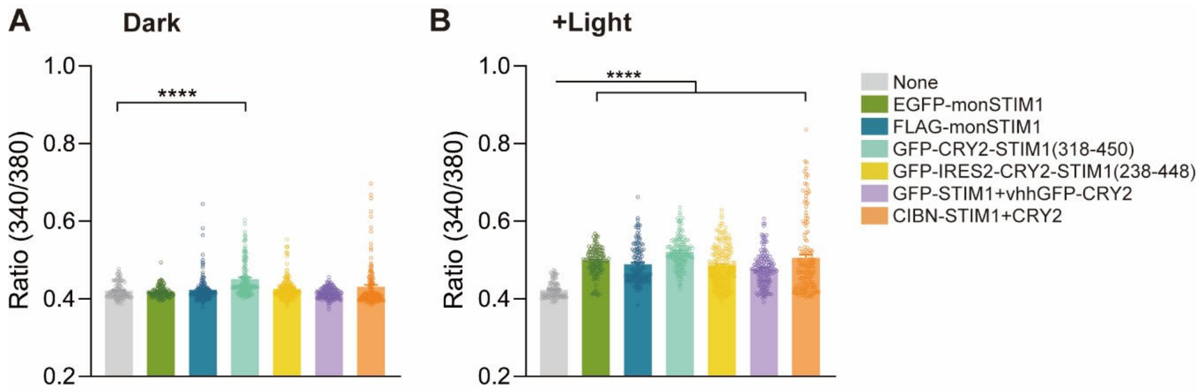

Figure S2. Measurement of relative Ca^{2+} levels by Fura-2 imaging in cells expressing monSTIM1 variants.

(A) Fura-2 ratio (emission 340 nm/380 nm) measured under dark conditions. **(B)** Fura-2 ratio measured after blue light illumination. EGFP-monSTIM1: n=120; FLAG-monSTIM1: n=150; EGFP-CRY2-STIM1(318-450): n=201; EGFP-IRES2-CRY2-STIM1(238-448): n=146; EGFP-STIM1+vhhGFP-CRY2: n=152; CIBN-STIM1+CRY2: n=144 cells. Data are presented as means ± SEM (*****p*<0.0001; Student two-tailed *t* test).

Primer list	Sequence
HA-F	5'-GACTGCTAGCGCCACCATGGGATACCCATACGACGTGCCTGACTACGCCCCACCGGTCATGAAGATGGAC-3'
HA-R	5'-GCATCCACGAGTGGGTACC-3'
FLAG-R	5'-GACTGCTAGCGCCACCATGGGAGACTACAAGGATGACGACGATAAGCCACCGGTCATGAAGATG-3'
LOV2-F	5'-GACTTGTACAAGGGCAGCCTGGCCACCACTCTAGAGCG-3'
LOV2-R	5'-GACTAAGCTTCAGCTCCTTGGCGGCCTC-3'
STIM1(336-486)-F	5'-GACTAAGCTTGAATCTCACAGCTCATGGTATGCTC-3'
STIM1(336-486)-R	5'-GACTGGATCCTTAAGACACAATCTCCTCATCCATGTCATC-3'
STIM1(238-448)-F	5'-GGAGGCTCCGGACTCAGATC-3'
STIM1(238-448)-R	5'-GACTGGATCCCTAGTGGATGCCAGGGTTGTTG-3'
STIM1(248-448)-F	5'-GACTTCCGGATTGGAGGGGTTACACCGAGC-3'
STIM1(336-448)-F	5'-GACTTCCGGAGAATCTCACAGCTCATGGTATGC-3'
STIM1(342-448)-F	5'-GACTTCCGGATATGCTCCAGAGGCCC-3'
STIM1(318-463)-F	5'-GACTTCCGGAGAGGAGGAGTTGG-3'
STIM1(318-463)-R	5'-GACTGGATCCCTAACTGCCCATCCA-3'
STIM1(318-450)-R	5'-GACTGGATCCCTACAGTGAGTGGATGC-3'
IRES2-F	5'-GATCGCTAGCGATCGATCGCGGCCGCATCCGCCCCTCTCCCTCC-3'
IRES2-R	5'-GATCTGTACACCATGGTTGTGGCCATATTATCATC-3'
CRY2-F	5'-GACTACCGGTCGCCACCATGAAGATGGACAAAAAGACCATCG-3'
CRY2-R	5'-GACTTGTACAATTCGTTGTCGAGGTCGGG-3'
vhhGFP-F1	5'-GACTGCTAGCGCCACCATGGTCCAACTGGTGGAGTCTGG-3'
vhhGFP-R1	5'-GACTACCGGTGGGCTTCCGCCGCTGGAGACGGTGACCTG-3'
vhhGFP-F2	5'-GACTTGTACAGCCACCATGGTCCAACTGGTGGAGTCTGG-3'
vhhGFP-R2	5'-GACTCTCGAGGGGCTTCCGCCGCTGGAGACGGTGACCTG-3'
AgeI-NheI-BamHI-HindIII oligomer-F	5'-CCGGGCTAGCGGTTCATCAGGTTCATCAGGATCC-3'
AgeI-NheI-BamHI-HindIII oligomer-R	5-AGCTGGATCCTGATGAACCTGATGAACCGCTAGC-3'
W3SL-F	5'-TGGATGGGCAGTTAGGGATCCCTCGAGATAATCAACCTCTGGATTACAAAATTTG-3'
W3SL-R	5'-TCCTGCGGCCGCTCGGTCCGTCCTGCGGCCGCTTTAAAAAAC-3'
GfaABC1D-F	5'-ATCGACGCGTAACATATCCTGGTGTGGAGTAGGGG-3'
GfaABC1D-R	5'-ATCGTCTAGAGCGAGCAGCGGA-3'

Table S1. **Oligos used in this study.**
